# High Drug Resistance Prevalence among Vertically HIV-Infected Patients Transferred from Pediatric Care to Adult Units in Spain

**DOI:** 10.1371/journal.pone.0052155

**Published:** 2012-12-17

**Authors:** Miguel de Mulder, Gonzalo Yebra, Adriana Navas, María Isabel de José, María Dolores Gurbindo, María Isabel González-Tomé, María José Mellado, Jesús Saavedra-Lozano, María Ángeles Muñoz-Fernández, Santiago Jiménez de Ory, José Tomás Ramos, África Holguín, Madrid Cohort of HIV-Infected Children

**Affiliations:** 1 HIV-1 Molecular Epidemiology Laboratory, Microbiology and Parasitology Department, Hospital Universitario Ramón y Cajal, IRYCIS and CIBER-ESP, Madrid, Spain; 2 Pediatrics Department, Hospital Universitario Infanta Leonor, Madrid, Spain; 3 Pediatrics Department, Hospital Universitario La Paz, Madrid, Spain; 4 Pediatrics Department, Hospital General Universitario Gregorio Marañón, Madrid, Spain; 5 Pediatrics Department, Hospital Universitario Doce de Octubre, Madrid, Spain; 6 Pediatrics Department, Hospital Carlos III, Madrid, Spain; 7 Molecular Immunobiology Laboratory, Hospital General Universitario Gregorio Marañón, Madrid, Spain; 8 Pediatrics Department, Hospital Universitario de Getafe, Madrid, Spain; University of California San Francisco, United States of America

## Abstract

**Background:**

Antiretroviral treatment (ART) has contributed to increased life expectancy of HIV-1 infected children. In developed countries, an increasing number of children reaching adulthood are transferred to adult units. The objectives were to describe the demographic and clinical features, ART history, antiviral drug resistance and drug susceptibility in HIV-1 perinatally infected adolescents transferred to adult care units in Spain from the Madrid Cohort of HIV-1 infected children.

**Methods:**

Clinical, virological and immunological features of HIV-1 vertically infected patients in the Madrid Cohort of HIV-infected children were analyzed at the time of transfer. *Pol* sequences from each patient were recovered before transfer. Resistance mutations according to the InternationaI AIDS Society 2011 list were identified and interpreted using the Stanford algorithm. Results were compared to the non-transferred HIV-1 infected pediatric cohort from Madrid.

**Results:**

One hundred twelve infected patients were transferred to adult units between 1997 and 2011. They were mainly perinatally infected (93.7%), with a mean nadir CD4+-T-cells count of 10% and presented moderate or severe clinical symptoms (75%). By the time of transfer, the mean age was 18.9 years, the mean CD4+T-cells count was 627.5 cells/ml, 64.2% presented more than 350 CD4+T-cells/ml and 47.3% had ≤200 RNA-copies/ml. Most (97.3%) were ART experienced receiving Highly Active ART (HAART) (84.8%). Resistance prevalence among pretreated was 50.9%, 76.9% and 36.5% for Protease Inhibitors (PI), Nucleoside Reverse Transcriptase Inhibitors (NRTI) and Non-NRTI (NNRTI), respectively. Resistance mutations were significantly higher among transferred patients compared to non-transferred for the PI+NRTI combination (19% *vs*. 8.4%). Triple resistance was similar to non-transferred pediatric patients (17.3% *vs.* 17.6%).

**Conclusion:**

Despite a good immunological and virological control before transfer, we found high levels of resistance to PI, NRTI and triple drug resistance in HIV-1 infected adolescents transferred to adult units.

## Introduction

By the end of 2010, of the 34 million people living with human immunodeficiency virus (HIV), there were 3.4 million children below the age of 15 years [1]. During 2010, 390.000 children were infected with HIV and 250.000 died from AIDS related causes [1]. In Western Europe and North America the HIV epidemic has remained stable since 2004. In 2010, one million infected individuals lived in Western and Central Europe, including 6,000 infected children [1]. In the WHO European Region of the 646 children who acquired the HIV infection through mother-to-child transmission (MTCT) [2], 19% of them originated from countries with generalized epidemics (in sub-Saharan Africa, the Caribbean and Asia).

Due to the expanded access to highly active antiretroviral treatment (HAART) and prevention efforts in HIV testing, prenatal care, formula feeding, elective Caesarean and pregnancy monitoring [3]–[5], few children were newly infected with HIV (<500) or died from AIDS-related illnesses (<500) in Western Europe in 2010. This reflects the extensive provision of services that can prevent MTCT of HIV [6], [7]. Despite the success of preventive measures, MTCT still occurs in high-income countries [8]–[10] mainly due to infected immigrants from countries with a high HIV prevalence and within social compartments that refuse pregnancy monitoring and HIV testing. A total of 80.827 cases of AIDS had been declared in Spain [11] by the end of 2010; these 958 were children infected through MTCT. During 2010, a total of 2907 new HIV infections cases were notified in Spain, twelve of them caused by MTCT (0.4%) mainly (8/12 cases) among foreign patients. In the region of Madrid, a total of 805 HIV infection cases were reported in 2010, 2 of them caused by MTCT [11].

In developed settings with access to HAART, perinatally acquired HIV-1 infection has become a chronic disease of childhood with increasing numbers of adolescents surviving to adulthood and transitioning from pediatric to adult services. Perinatally infected adolescents have been heavily pretreated, have a long history of treatment with many switches and variable levels of adherence to the treatment have been reported. Sub-optimal treatments and non-complete compliance can increase the prevalence of drug resistance mutations in HIV thus, compromising the success of present and future treatment options.

Successful transition to adult services has become a necessity in these heavily pretreated patients. Teenagers growing up with HIV/AIDS have common problems related to social difficulties and to side-effects of HIV and HAART which play an impact on their growth and development. The objectives of this study were to describe the demographic and clinical features, antiretroviral therapy (ART) history, antiviral drug resistance and susceptibility to drugs in HIV-1 perinatally infected adolescents transferred to adult units in Spain from the Madrid Cohort of HIV-1 infected children.

## Patients and Methods

### Study Population

Since the beginning of the HIV epidemic in Spain, a total of 534 patients have been registered in the Madrid cohort of HIV-infected children established in 2003. By the end of December 2011, 175 of them still remained under clinical follow-up in pediatric units, 112 had been transferred to adult units, 62 had been lost to follow-up and 185 had died. In this study we selected the 112 patients from the cohort that had reached adolescence and been transferred to adult units in 8 public hospitals from 1997 to December 2011. Clinical and epidemiological features of all transferred patients were recorded from the database of the cohort.

An additional cohort of HIV-1 infected patients was used to compare results on drug resistance and drug sensitivity. The selected cohort consisted of the HIV Madrid cohort of non-transferred perinatally infected patients previously described [12], [13].

This study was part of a project approved by the review board of the Hospital Universitario Ramón y Cajal Clinical Research Ethical Committee. It was designed to protect the right of all subjects involved under the appropriate local regulations. To maintain subject confidentiality, a unique number was assigned to each specimen, and written consent obtained for each patient by clinicians.

### Drug Resistance Analysis

For the drug resistance study, we selected those transferred patients according to *pol* sequence, genotypic resistance profile or sample availability by December 2011. Most genetic sequences and genotypic resistance profiles were previously reported [12], [13] or recovered from clinical routine drug-resistance tests performed in hospitals where patients were or had been under follow-up. When more than one sample was available per patient, we selected the closest and previous to the time of transference to adult units among transferred subjects and the most recent for non-transferred patients.

Previously reported genotypes were performed from infected samples (immortalized DNA, plasma or peripheral blood mononuclear cells, PBMCs) kindly provided by the HIV BioBank integrated in the Spanish AIDS Research Network (RIS) [14], [15]. Samples from patients were processed following current procedures and frozen immediately after their reception. Sample collection, processing and storing were performed under international guidelines for biological storing and under supervision of a Scientific and Ethical Committee. HIV-1 subtyping of new sequences was performed by phylogenetic analysis (phy) as previously described [13].

The prevalence of transmitted drug resistance among naïve patients was defined according to the list of mutations for Transmitted Drug Resistances (TDR) surveillance, as recommended by the WHO [16] using the Calibrated Population Resistance tool [17]. Drug-resistance mutations (DRM) in pretreated patients were defined by the International AIDS Society-USA list (IAS) [18]. Drug susceptibility was estimated for each available antiretroviral according to the HIVdb Interpretation Algorithm version 6.0.11 (Stanford University, Palo Alto, CA, USA) [19].

### Statistical Analysis

Prevalence was expressed in percentage with a 95% confidence interval (CI). CI tests were performed with Epidat 3.1 (Pan American Health Organization). Statistical significance was set at p<0.05.

## Results

### Baseline Features of the Transferred Population

A total of 112 patients of the Madrid cohort of HIV-infected children transferred from pediatric services to adult units in different hospitals in Madrid between 1997 and December 2011 were selected for this study. Baseline characteristics of the non-transferred (n = 131), total transferred (n = 112), and transferred patients with available genotypic profile (n = 63) are summarized in **Table 1**. All transferred patients were HIV-1 diagnosed at childhood (mean 2 years of age), the majority were born in Spain (91.9%) and mainly infected through MTCT (93.7%). Only a few (12.5%) were adopted. Most (81.3%) were diagnosed along the 1985–1994 period. The median duration of follow-up was 13.2 (Standard Deviation, SD 5.2), 15.6 (SD 4.5), and 16.7 (SD 3.6) years for non-transferred, transferred (n = 112) and transferred with available resistance genotypic profile, respectively.

**Table 1:** Baseline characteristics of the non-transferred, transferred and transferred with available genotypic profile patients.

Features	Non-transferred n = 131	Transferred n = 112	Transferred with genotype* n = 63
	[n (%)]	[n (%)]	[n (%)]
Adopted	31 (23.7)	14 (12.5)	8 (12.7)
Female gender	76 (58)	60 (53.6)	34 (54)
Median age until diagnosis (years)	0.5	2	1.4
Non-B variants prevalence (%)	11.6	–	1.9
*Demographics*			
Caucasian	100 (76.3)	98 (87.5)	59 (93.6)
Hispanic	6 (4.6)	4 (3.5)	2 (3.2)
Romani	4 (3.1)	3 (2.7)	1 (1.6)
African**	18 (13.7)	2 (1.8)	–
Other	2 (1.5)	2 (1.8)	1 (1.6)
Unknown	1 (0.8)	3 (2.7)	–
*Origin* a			
Europe	112 (85.5)	105 (93.7)	61 (96.8)
North America	1 (0.8)	–	–
South and Central America	8 (6.1)	5 (4.5)	2 (3.2)
North Africa	2 (1.5)	–	–
Sub-Saharan Africa	7 (5.3)	2 (1.8)	–
Asia	1 (0.8)	–	–
*Year of HIV diagnosis*			
1985–1989**	1 (0.8)	31 (27.7)	16 (25.4)
1990–1994**	37 (28.2)	60 (53.6)	39 (61.9)
1995–1999**	54 (41.2)	18 (16)	7 (11.1)
2000–2004**	28 (21.4)	3 (2.7)	1 (1.6)
2005–2009	10 (7.6)	–	–
Unknown	1 (0.8)	–	–
*Route of infection*			
Perinatally	127 (96.9)	105 (93.7)	61 (96.8)
Transfusion	3 (2.3)	5 (4.5)	2 (3.2)
Unknown	1 (0.8)	2 (1.8)	–
*Year of transfer*			
1997–1999	–	3 (2.7)	–
2000–2002	–	9 (8)	1 (1.6)
2003–2005	–	27 (24.1)	13 (20.7)
2006–2008	–	33 (29.5)	21 (33.3)
2009–2011	–	40 (35.7)	28 (44.4)
*Nadir CD4 count achieved*		Mean 10%	Mean 11%
<15%	67 (51.1)	75 (67)	43 (68.3)
15–24%	42 (32.1)	24 (21.4)	13 (20.6)
≥25%	19 (14.5)	13 (11.6)	7 (11.1)
Unknown	3 (2.3)	–	–
*Nadir CD4 count achieved (cells/mm^3^)*			
<200**	35 (26.7)	64 (57.1)	31 (52.4)
200–499	63 (48.1)	37 (33)	26 (38.1)
≥500**	30 (22.9)	11 (9.9)	6 (9.5)
Unknown	3 (2.3)	–	–

a Origin of patients by country: Spain (n = 103), Portugal (n = 1), Romania (n = 1), Honduras (n = 2), Argentina (n = 1), Mexico (n = 1), Peru (n = 1), Cape Verde (n = 1), Equatorial Guinea (n = 1).

* Transferred to adult units with available resistance genotyping profile.

** Statistical differences (p<0.05) have been found between transferred and non-transferred patients for these features. HIV-1 non-B variants include HIV-1 non-B subtypes and recombinants.

Advanced stages of immunosuppression were observed as a result of the long term infection and scarce effective antiretroviral availability before 1996. Over two thirds of transferred patients reached less than 15% CD4+ cell counts and half (57.1%) reached <200 cells/mm^3^. The mean nadir CD4+ T-cells count was 10%. Monotherapy was the first ARV treatment in 59.8%, mainly with AZT (79.1%), 23.2% started with combined therapy (including AZT backbone in 88.4% of them) and only 14.3% with HAART.

In the transferred cohort compared to the non-transferred, a statistical significant lower number of African patients were found (1.8% *vs.* 13.7%, p<0.05), a significant higher number of children reached nadir CD4-Tcell values below 200 (57.1 *vs.* 26.7, p<0.05) and a lower number of patients achieved CD4 T-cells over 500 (9.9% *vs.* 22.9%, p<0.05). No statistical differences were found in the baseline studied characteristics between transferred patients with and without available genotype (**Table 1**).

### Features of the Population at the Time of Transfer

Characteristics of the study population at the time of transfer to adult units are shown in **Table 2**. By the time of transfer, the mean age was 18.9 years and the mean CD4+T-cells count 627.5 cells/ml. 5.4% presented less than 15% CD4+T-cells, 66% more than 25% and 55.3% more than 500 CD4+ cells/ml counts. Nearly all (98.2%) had presented signs and symptoms of AIDS according to CDC classification [20], which were severe in 34.8% of cases. Immunological status at the time of the transfer revealed an immunologically severe suppression in 66.9% (CDC stage 3). Among the 102 patients with available viral load data, 56.2% had ≤500 RNA-copies/ml and 38.4% undetectable viraemia (≤50 RNA-copies/ml).

**Table 2:** Characteristics of the non-transferred, transferred and transferred with available genotypic profile patients by December 2011.

Features	Non-transferred n = 131	Transferred n = 112	Transferred with genotype* n = 63
*Mean age (years)*	14.7	18.9	18.5
*LTNP [n (%)]*	1 (0.8)	5 (4.5)	2 (3.2)
*Median ART duration [years (SD)]*	12 (4.5)	11.5 (4.8)	13.5 (4.1)
*Median follow-up [years SD)]*	13.2 (5.2)	15.6 (4.5)	16.7 (3.6)
*Immunological status [n (%)]*			
1	8 (6.1)	4 (3.6)	2 (3.2)
2	41 (31.3)	31 (27.7)	18 (28.5)
3	79 (60.3)	75 (66.9)	43 (68.3)
Unknown	3 (2.3)	2 (1.8)	-
*Clinical status [n (%)]*			
N	-	2 (1.8)	1 (1.6)
A**	49 (37.4)	26 (23.2)	14 (22.2)
B**	35 (26.7)	45 (40.2)	23 (36.5)
C	43 (32.8)	39 (34.8)	25 (39.7)
Unknown	4 (3.1)	-	-
*CD4 count (%) [n (%)]*			
<15%	3 (2.3)	6 (5.4)	3 (4.8)
15–24%	18 (13.7)	26 (23.2)	15 (23.8)
≥25%**	105 (80.2)	74 (66)	43 (68.2)
Unknown	5 (3.8)	6 (5.4)	2 (3.2)
*CD4 count (cells/ml) [n (%)]*			
	Mean 770.5 cells/ml	Mean 627.5 cells/ml	Mean 654 cells/ml
≤200	3 (2.3)	4 (3.6)	1 (1.6)
201–350	6 (4.6)	7 (6.3)	5 (7.9)
351–500	13 (9.9)	10 (8.9)	4 (6.4)
>500**	99 (75.6)	62 (55.3)	36 (57.1)
Unknown	10 (7.6)	29 (25.9)	17 (27)
*Viral load (HIV-1 RNA-copies/ml) [n (%)]*			
≤20**	41 (31.3)	12 (10.7)	9 (14.2)
21–50	44 (33.6)	31 (27.7)	23 (36.5)
51–200	10 (7.6)	10 (8.9)	4 (6.4)
201–500	7 (5.3)	10 (8.9)	2 (3.2)
501–1,000	-	4 (3.6)	3 (4.8)
1,001–10,000	12 (9.2)	19 (17)	9 (14.3)
>10,000	13 (9.9)	16 (14.3)	11 (17.4)
Unknown	4 (3.1)	10 (8.9)	2 (3.2)
*ART experience [n (%)]*			
Drug naive	1 (0.8)	3 (2.7)	2 (3.2)
PI-experienced	116 (88.6)	95 (84.8)	54 (85.7)
NRTI-experienced**	130 (99.2)	106 (94.6)	59 (93.6)
NNRTI-experienced	99 (75.6)	81 (72.3)	47 (74.6)
FI-experienced	-	3 (2.7)	1 (1.6)
InI-experienced	-	1 (0.9)	1 (1.6)
PI+NRTI+NNRTI-experienced	88 (67.2)	72 (64.3)	42 (66.7)
*Treatment status [n (%)]*			
HAART**	124 (94.6)	95 (84.8)	54 (85.7)
Stopped-treatment	3 (2.3)	11 (9.8)	6 (9.5)
Naive	1 (0.8)	3 (2.7)	2 (3.2)
Monotherapy	1 (0.8)		
Combined	2 (1.5)	3 (2.7)	1 (1.6)

SD, standard deviation; ART, antiretroviral therapy; PI, protease inhibitors; NRTI, nucleoside reverse transcriptase inhibitors; NNRTI, non-NRTI; FI, fusion inhibitors, InI; integrase inhibitors.

* Transferred to adult units with available resistance genotyping profile.

** Statistical differences (p<0.05) have been found between transferred and non-transferred patients for these features.

Comparison among transferred and non-transferred patients by December 2011 revealed that transferred patients had a worst immunological-virological profile comparing to the non-transferred group. A lower number of transferred patients were categorized in clinical status A (23.2% *vs.* 37.4%, p<0.05) and achieved undetectable levels of viraemia (38.4% *vs.* 64.9%, p<0.05). T-cell CD4 counts (either ≥25% or >500 CD4+ T-cells) were also lower among transferred and a lower number of transferred were on HAART (84.8% *vs.* 94.6%, p<0.05). No statistical differences were found in studied clinical features by December 2011 between transferred patients with and without available genotype (**Table 2**).

### ART Experience among Transferred Patients

The transferred cohort started any type of ART with a median age of 5.6 years (SD 3.5 years) and the median duration of ART was 11.5 years (SD 4.8 years). Only 3 (2.7%) of the 112 transferred individuals remained drug naïve at transfer and the rest (97.3%) were ART experienced. Most (84.8%) were receiving HAART, 9.8% had stopped treatment and 2.7% were receiving combined therapy at the transfer time according to clinical reports. The most commonly used HAART combinations in our cohort were 2NRTI+1NNRTI (32.6%) and 2NRTI+1PI (28.4%). The pretreated patients presented a long treatment history and had experienced several different ART combinations; the mean number of regimens was five, with at least 3 HAART regimens in 48% of them. The main ART families were NRTI, PI and NNRTI, 94.6%, 84.8%, and 72.3% respectively (**Table 2**). Triple class experience was found in almost two-thirds of patients. Use of other drug families was scarce and only three transferred patients had experience with the fusion inhibitor, enfuvirtide and one of them also had received raltegravir, an integrase inhibitor. Adherence history was assessed according to clinical charts data but was not available in all cases.

### High Prevalence of HIV-1 Resistant Variants in Pretreated Transferred Patients

Among the 112 patients transferred before the end of December 2011, only in 63 (56.2%) subjects drug resistance genotypes could be analyzed. Among them, in 48 (76.2%) cases the *pol* sequence had been previously reported by our group [12], [13]. In the remaining 15 patients, six patients had an available plasma specimen stored in the HIV-1 Spanish Biobank and new HIV-1 *pol* sequences were newly generated as previously reported [13]. Other 9 patients presented a genotypic resistance profile derived from *pol* sequences obtained from plasma specimens recovered from clinical routine drug-resistance tests performed in hospitals where the patients were or had been under follow-up. However, no fasta format sequences of any profile were available. Clinical specimens in all 63 genotyped samples were obtained before the transfer time to adult units: during 1993–1999 (5 genotypes), 2000–2004 (27 genotypes) and 2005–2010 (31 genotypes). The median age of patients when genotypic data was generated was 14.7 years old (SD 4.4). The 63 patients with available genotypic data included five genotypes (accession numbers HQ426734, HQ426806, HQ426860, HQ426867, HQ426893) performed before treatment was initiated. Out of these five, only 2 patients remained drug naïve until they were transferred to adult units while the other 3 received any ART during their follow-up. None of these sequences harboured TDR mutations.

Among the 63 transferred patients of the Madrid cohort of HIV-infected children with available *pol* sequences or resistance profiles, the prevalence of HIV drug resistance mutations was analyzed according to the drug class family. Fifty-eight were pretreated and 5 remained drug-naïve at sampling time. No TDR mutations were found among the *pol* sequences from the 5 transferred naïve subjects. The most prevalent DRM found in the 58 transferred ART-experienced patients were: for PI, L90M (27.5%), V82A (15.7%), M46I (13.7%) and D30N (9.8%); for NRTI, M41L (48.1%), D67N (40.4%), T215Y (40.4%), L210W (34.6%), M184V (25%), T69D (23.1%), and K219Q (23.1%); and for NNRTI, K103N (19.2%), Y181C (7.7%) and G190A (7.7%).

Global resistance prevalence among the 58 transferred ARV-exposed pretreated patients with available *pol* sequences or resistance profiles was 50.9% for PI, 76.9% for NRTI and 36.5% for NNRTI (**Table 3**). No primary drug resistance mutations were found among naïve subjects. According to our data, no statistical differences were found between the resistance rate and the duration of treatment (data not shown).

**Table 3:** Comparison of HIV drug resistance mutations prevalence according to drug class family in the non-transferred and the transferred pediatric cohorts.

	Transferred patients^a^ (n = 58)	Non-transferred children^b^ (n = 131)
Patients with available PR	51	125
Patients with available RT	52	116
Prevalence of drug resistance mutations (%) [95% CI]^c^		
Global (to any class)	81.0 [70.1–92]	69.5 [61.2–77.7]
To PIs	50.9 [36.3–65.7]	36.8 [27.9–45.7]
To NRTIs	76.9 [64.5–89.3]	62.1 [52.8–71.3]
To NNRTIs	36.5 [22.5–50.6]	40.5 [31.2–49.9]
To NRTI+NNRTI	12.1 [2.8–21.3]	12.2 [6.2–18.2]
To PI+NRTI**	19 [8–29.9]	8.4 [3.3–13.5]
To PI+NNRTI	–	0.8 [0.02–4.2]
To PI+NRTI+NNRTI	17.3 [6.7–27.8]	17.6 [10.7–24.5]

a Selected patients from the Madrid cohort of HIV-1 infected children that have been transferred to adult units by December 2011.

b Pretreated patients selected from the Madrid cohort of HIV-1 infected children excluding those transferred to adult units.

c Prevalence of drug resistance mutations was determined following the IAS-USA 2011 list [18]. PR, protease; RT, reverse transcriptase; NRTI, nucleoside reverse transcriptase inhibitors; NNRTI, non-NRTI; PI, protease inhibitors.

** Statistical difference (p<0.05) has been found between transferred and non-transferred patients for this feature.

### Higher Prevalence of Drug Resistance Mutations Among Transferred Patients than in Non-transferred

Besides assessing the global prevalence of DRM in the 58 pretreated patients of the transferred cohort, prevalence of DRM was also compared to the non-transferred cohort. The first included the 58 transferred and pretreated patients with available genotypic data compared to the 131 non-transferred pretreated pediatric patients from the Madrid cohort of HIV-1 infected children (**Table 3**). All 131 non-transferred pediatric patients had available resistance genotype, previously reported [12], [13] and with available GenBank accession numbers.

Transferred patients tended to present higher DRM prevalence when compared to non-transferred children (**Table 3**). Drug resistance mutations were found in a higher number of transferred patients than in the non-transferred pretreated population under follow up in Madrid for PI (50.9% *vs*. 36.8%, p = NS) and for NRTI, 76.9% *vs*. 62.1%, p = NS) and lower for NNRTI (36.5% vs. 40.5%, p = NS). However, resistance prevalence was significantly higher among transferred patients for the PI+NRTI combination (19% *vs*. 8.4%, p<0.05). Triple resistance was similar to non-transferred pediatric patients (17.3% *vs.* 17.6%, p = NS).

### Higher Predicted Level of Resistance to PI and NRTI among Transferred Patients

**Figure 1:**
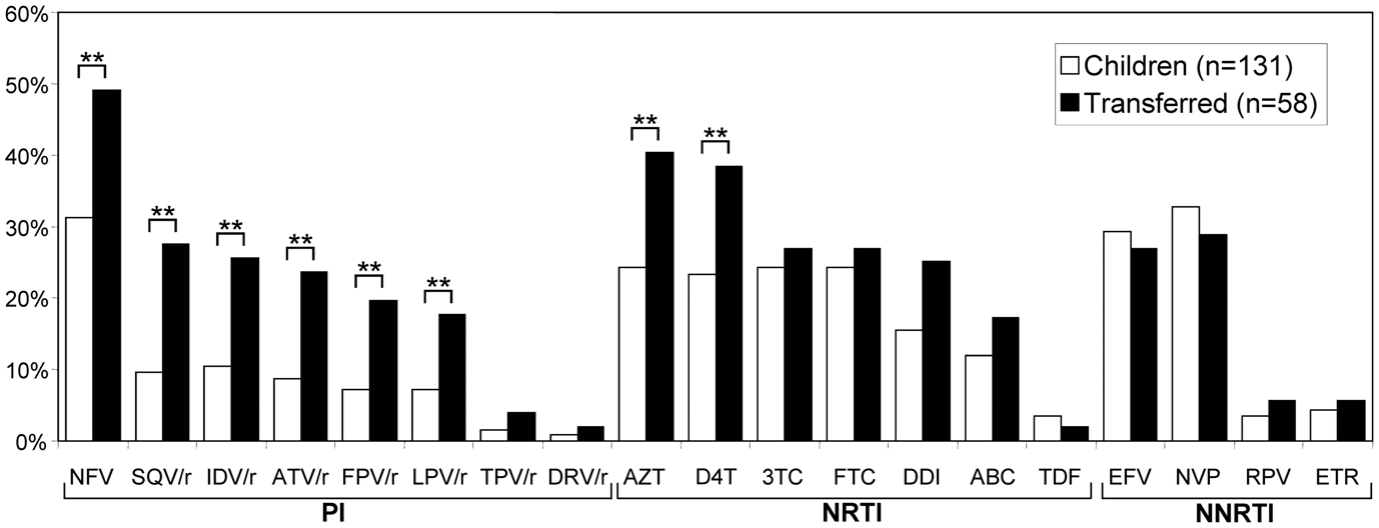
Predicted resistance level to antiretroviral drugs in pretreated patients from the two studied cohorts. Resistance level was estimated according to the HIVdb Interpretation Algorithm (Stanford University, Palo Alto, CA, USA) [19]. PI, protease inhibitors: nelfinavir (NFV), saquinavir/r (SQV/r), indinavir/r (IDV/r), atazanavir/r (ATV/r), fosamprenavir/r (FPV/r), lopinavir/r (LPV/r), tipranavir/r (TPV/r) and darunavir/r (DRV/r), where “/r” indicates co-administration with low-dose ritonavir (RTV) for pharmacological “boosting”. NRTI, nucleoside reverse transcriptase inhibitors: zidovudine (AZT), stavudine (d4T), lamivudine (3TC), emtricitabine (FTC), didanosine (DDI), abacavir (ABC), tenofovir (TDF). NNRTI, non-nucleoside reverse transcriptase inhibitors: efavirenz (EFV), nevirapine (NVP), rilpivirine (RPV), etravirine (ETR). **Statistical differences (p<0.05) in resistance levels have been found between transferred and non-transferred patients for these drugs.

**Figure 1** shows the comparison of the predicted level of resistance to each drug in all pretreated patients from the two study cohorts. Analysis of the genotypic resistance interpretation revealed that transferred adolescents presented a significantly higher predicted level of resistance to all drugs from the PI and NRTI families probably explained by the long-term therapy history of these patients. Half of the transferred patients carried infecting HIV-1 variants resistant to NFV, followed by AZT and d4T (nearly 40% of them). Similar predicted resistance was observed for 3TC and FTC in the non-transferred and transferred study groups (around 20%). Between nearly 20% and 30% of the adolescents in Madrid were infected with variants resistant to the remaining PI drugs, except for TPV/r and DRV/r, the new PI drug generation. EFV and NVP were the NNRTI with the most compromised susceptibility in both cohorts (around 20–30%), being higher in non-transferred patients. Data revealed a low (2–5%) predicted resistance level to etravirine and rilpivirine, the new NNRTI drugs; resistance levels to these drugs remained low in a gap between 2% and 5% for both studied cohorts.

### Low Prevalence of HIV-1 Non-B Variants in Transferred Patients

Most (98.1%) of the transferred patients were infected by subtype B, the most prevalent HIV-1 variant in North America and Western Europe, including Spain [13], [21]. Only one transferred perinatally infected female carried a “pure” sub-subtype A2. She was a white-Caucasian, vertically infected and born of Spanish parents in 1992. Prevalence of HIV-1 non-B variants among the 54 transferred patients with available *pol* sequence was low (1.9%, 1/54), compared to the 11.5% (15 patients) found among the 131 non-transferred children of the same pediatric cohort (**Table 1**) or the 12.2% reported n the Spanish cohort of antiretroviral treatment-naïve HIV-infected patients [21]. Of interest, recombinant viruses were absent in transferred patients although found in 60% (9/15) of infections caused by non-B variants in the non-transferred pediatric cohort, respectively.

### Sequence Data

*Pol* (PR and/or RT) sequences from 48 (88.8%) of the 54 transferred patients included in this study had been previously submitted to GenBank [13]: HQ426715, HQ426719, HQ426725, HQ426728, HQ426734, HQ426766, HQ426768, HQ426779, HQ426780, HQ426788, HQ426799, HQ426806, HQ426807, HQ426818, HQ426826, HQ426840, HQ426842, HQ426847, HQ426850, HQ426857, HQ426860, HQ426861, HQ426866, HQ426867, HQ426868, HQ426869, HQ426874, HQ426879, HQ426880, HQ426883, HQ426889, HQ426890, and HQ426893. In reference [12]: JQ351951, JQ351960, JQ351984, JQ351986, JQ351988, JQ351989, JQ351995, JQ351997, JQ352005, JQ352006, JQ352010-JQ352012, JQ352014, and JQ352021. The 6 newly generated *pol* sequences for this study were submitted to GenBank with the following accession numbers: JQ828989-JQ828994. GenBank accession numbers for the 131 sequences from non-transferred children were previously reported [12], [13].

## Discussion

Due to the low number of new cases of HIV infection caused by MTCT in developed countries [2], perinatally infected cohorts will tend to reduce. However, several studies have assessed the current state of adolescent survivors of perinatally or early acquired HIV infection [22]–[33]. As ART becomes more widely available in the developing world, increasing the effective viral suppression with immunologic reconstitution, we can expect a steady increase in the number of children being transferred into adult units, as reported in European [24]–[26] and North American pediatric [22], [29], [32] cohorts. This recent process described in high income regions could eventually occur in developing countries, if the access to optimal treatment is maintained.

### High Rate of Perinatally HIV-1 Infected Patients Transferred to Adults Units in Madrid

Features of the transferred patients from the Madrid cohort of HIV-1 infected children have been scarcely studied [34]. Among the 534 patients from the Madrid cohort of HIV-1 infected children by the end of December 2011, a total of 112 (21%) had reached adolescence and were transferred to adult units from 1997 through 2011. The first patient was transferred in 1997. The high percentage of transferred patients, even higher than in the British pediatric HIV CHIPS cohort [23] (n = 103; 16% *vs*. n = 112; 21%), is directly related to the high number of HIV-1 infections occurring in Spain in the early nineties due to the so called “heroin epidemic”. The abuse of heroin during the 1980s and 1990s in Spain had a special impact in women, reaching the highest AIDS incidence and prevalence in Western Europe, which led to a high incidence of MTCT in children born between 1980 and 1990 [11], [35]. Future transitioning programs will include a smaller number of patients due to the low number of new diagnoses [2], [9] of HIV-1 among children in high income countries.

### Higher Prevalence of Drug Resistant Strains among Transferred and Non-transferred Patients

The emergence of drug resistance due to incomplete viral suppression and incomplete adherence are the major obstacle for an effective ART [36]. Adolescents with perinatally acquired HIV are heavily pretreated, have a long history of treatment with many regimen switches, and present variable levels of adherence to the treatment, mainly during adolescence. However, there are few data about disease progression, response to ART and drug resistance prevalence in vertically HIV-infected adolescents in the era of effective therapy, even though their number is increasing in developed countries where ARV therapy is guaranteed. The results presented showed that two thirds of transferred patients from the Madrid cohort of HIV-1 infected children to adult units were triple class-experienced, higher than in other cohorts as in the UK (64.3% *vs*. 47%, respectively) [23]. Results may be explained by longer exposure to older, less efficacious treatments. In fact, HIV infected patients during childhood in our cohort were mainly infected during the early 1990’s, and had to face the monotherapy and dual therapy regimens available at the time, thus increasing the risk of virological failures due to resistance development. The first patient reached adulthood in 1997 and thus had received previous, less efficacious treatments during his childhood.

Interestingly, DRM prevalence to all 3 drug classes among transferred patients was higher than for non-transferred infected children. This fact could be caused by regimen switches due to therapeutic failure or because of the availability of new drugs during the infection period. As a consequence of their long treatment history and the treatment switches they have experimented, 81% of the patients transferred to adult units harboured resistant virus to at least one of the drug classes, higher than in non-transferred children (69.5%) mainly for NRTI, the first available drug class for clinical use, and for PI. This high rate of resistance could have compromised the susceptibility found in our data from both the PI and NRTI families.

In fact, the pediatric population (transferred and non-transferred) infected by viruses carrying triple resistance mutations was significantly higher than in pretreated adults from Madrid (17% *vs*. 8.6%), and moreover, was higher than in adolescents from the UK and Ireland (12%) [23] and the COHERE pediatric cohort of perinatally infected children in Western Europe aged less than 16 years (10%) [37]. On the other hand, DRM to NNRTI was slightly higher in the Madrid cohort of HIV-1 infected children than in the transferred group, so reflecting their preferential use in the pediatric population during the study period. These results on prevalence highlight the potential problem that clinicians have and will face in the near future with HIV-1 adolescents who are highly resistant to all drug classes.

### Possible Candidates to Rescue Transferred Highly Pretreated Patients

Treatment failure in children during ART is frequent, develops fast and with more extensive drug resistance than in adults, leading to detectable viral loads and immunological damage [37]–[42]. Thus, keeping a close surveillance of adherence has to be a priority in these heavily pretreated adolescents requiring treatment for life [42], [43]. Moreover resistance studies are also required to optimize ARV regimens. According to the predicted drug susceptibility, our data revealed than TDF (NRTI) and the new PI (TPV/r and DRV/r) and NNRTI (ETR and RPV) drugs could be good alternatives for inclusion in future ARV regimens to control the viraemia in highly pretreated transferred adolescents in Madrid. Adolescents could also benefit from the newly licensed drugs to treat HIV-1 infection for adults. Other drug families (cell-entry and integrase inhibitors), could be good candidates to control viraemia among pretreated transferred patients in Madrid due to their previous scarce exposure (<1%). However, the presence of X4-tropic variants in over 80% of the cohort of antiretroviral-experienced children and adolescents with vertical HIV-1 infection in Madrid has recently been reported [44]. The authors indicate a very limited role for CCR5 antagonists as part of salvage regimens for highly treatment-experienced vertically infected patients with extensive antiretroviral drug resistance [44]. Thus, integrase inhibitors could be the best rescue alternative in the cases of therapeutic failure with multiresistance, although additional genetic resistance studies should be performed to guarantee their usefulness.

### Low Prevalence of HIV-1 Non-B Variants Infection among Transferred Patients

Epidemiological differences related to the nature of HIV-1 infecting variants were found between the non-transferred cohort of HIV infected children (11.4%) and with those transferred to adult units (1.9%). Pediatric patients that reached adulthood and were transferred to adult units were mainly infected by subtype B (98.2%). This fact is explained by the long term infection of our patients (mean age of 18.9 years), reflecting the local epidemiological situation in Spain at the time in which circulating variants other than B had not yet been detected in Spain.

Previous studies in the Madrid cohort of HIV-1 infected children and adolescents reported a non-B prevalence of 10% [12], [13]. In fact, in Madrid an increase of HIV-1 non-B infections after year 2000 was reported among infected children [12] and among newly HIV infected adults [14]. Interestingly, the increasing complexity of the epidemic reported in HIV-infected adults [21], [45] and in the pediatric population in Madrid [13] was not observed among the transferred population, in whom no recombinant strains were found. As a limitation, only half (n = 54) of the transferred cohort had an available *pol* sequence to perform HIV-1 variant characterization. Prevalence of HIV-1 non-B variants infecting children and adults from Madrid has been estimated as about 10% [12], [13], [21]. Similar results have been published for other pediatric cohorts, where non-B infections ranged from 5 to 15% [46], [47].

### Conclusions

Understanding the progress of HIV-1 infected children through pediatric care until they reach adolescence in developed countries could help to improve and plan adequate clinical and psychological transitioning services for the HIV-1 infected children that will reach adolescence [23], [24], [26], [33] in the future. The increasing resistance prevalence among the HIV-infected-pediatric population in Spain highlights the importance of specific drug-resistance and drug-susceptibility surveillance in long-term pretreated children to optimize treatment regimens. Clinicians in Spain should consider that young adults infected during childhood do not present same clinical features as those young adults infected by other routes and that they require a specific clinical follow up.
